# The Two Revenges

**Published:** 1879-09

**Authors:** 


Some centuries since, the chief of the district, Maclean of Lochbuy, had a grand hunting excursion. To grace the festivity, his lady attended, with his only child, an infant, then in the nurse's arms. The deer, driven by the hounds, and hemmed in by surrounding rocks, flew to a narrow pass, the only outlet they could find. Here the chief had placed one of his men to guard the deer from passing; but the animals rushed with such impetuosity, that the poor forester could not withstand them. In the rage of the moment Maclean threatened the man with instant death; but his punishment was commuted to a whipping, or scourging in the face of the clan, which in those feudal times was considered a degrading punishment, fit only for the lowest of menials, and the worst of crimes. The clansman burned with anger and fierce revenge. He rushed forward, plucked the tender infant, the heir of Lochbuy, from the hands of the nurse, and bounding to the rocks, in a moment stood on an inaccessible cliff, projecting over the water. The screams of the agonized mother and chief at the awful jeopardy in which their only child was placed, may easily be conceived. Maclean implored the man to give him back his son, and expressed his deep contrition for the degradation he had in a moment of excitement inflicted on his clansman. The other replied that the only conditions on which he would consent to the restitution were, that Maclean himself should bare his back to the cord, and be publicly scourged as he had been. In despair the chief consented, saying he would submit to any thing, if his child were but restored. To the grief and astonishment of the clan, Maclean bore this insult, and when it was completed, begged that the clansman might return from his perilous situation with the young chief. The man regarded him with a smile of demoniac revenge, and, lifting high the child in the air, plunged with him into the abyss beneath. The sea closed over them, and neither, it is said, ever emerged from the tempestuous whirlpools and basaltic caverns that yawned around them.

